# Note from the Editor: Revised Instructions to Authors

**Published:** 2010-08

Authors planning to submit manuscripts to *Environmental Health Perspectives* (*EHP*) should note that we have revised our Instructions to Authors. The revised Instructions to Authors are included at back of this issue and are available on the journal’s website (http://www.ehponline.org/). Four points bear special attention.

First, over the last several months, it is has become clear that the journal needed to provide more guidance concerning reviews. Please note that the journal now has three categories of review papers:

- **Substantive Reviews** provide an overview, integration of information, and critical analysis of a particular field, research, or theme related to environmental health sciences. The strengths and weaknesses of individual studies and weight of evidence should be discussed. In addition, we encourage authors to identify research gaps and make recommendations for future research.
- **Quantitative Reviews and Meta-Analyses** present and contrast—and when appropriate, combine—data across studies to address a specific question related to environmental health sciences. Authors should provide inclusion criteria and strategies used to search the literature, and discuss strengths and weaknesses of studies as well as potential causes of discordant findings. As in the case of Substantive Reviews, we encourage identification of research gaps and recommendations.
- **Emerging Issue Reviews** identify emerging ideas, concepts, or trends in the area of environmental health sciences. They should have a highly focused narrative and a limited set of references. Emerging Issue Reviews, limited to 5,000 words, undergo an expedited review process.

Second, authors should be aware that the journal is placing greater emphasis on word limits for submissions. Papers exceeding the word limits described in the Instructions to Authors will be returned to the authors before being considered for peer review. We suggest placing some types of materials, such as lengthy descriptions of previously published methods, into Supplemental Material. However, a brief description of methods in the main body of the manuscript is required. Because references contribute considerably to the length of most papers, authors should include only the most relevant citations.

Third, each manuscript is now routinely checked for possible plagiarism before peer review. Definitions of four common kinds of plagiarism are described in the *American Medical Association Manual of Style: A Guide for Authors and Editors*, 10th edition (New York:Oxford University Press, p. 158).

Finally, a number of readers have recommended that abstracts should contain a clear statement about the potential impact of the research findings on the area of environmental health. Authors are encouraged to include a statement about the impact of their research in the Conclusion or Relevance section of their abstract.

